# To Mosquitoes, People with Malaria Smell Like Dinner

**DOI:** 10.1371/journal.pbio.0030306

**Published:** 2005-08-09

Malaria is a misnomer. People used to believe that poisoned or “bad air,” the translation of the Italian phrase “mal aria,” caused disease. In the 19th century, when parasitologists figured out that single-celled parasites cause malaria, they didn't bother to change the disease's name. Experimenters proved that these parasites need a host organism to survive—so they can't be transmitted through air—and that the hosts, mosquitoes, carry the parasite to humans. Researchers were optimistic that if they could find a disease's cause, they could also find the cure. Kill the mosquitoes and eradicate malaria. And with the advent of DDT and less environmentally harmful insecticides, potent anti-malarial drugs, and international funding in the late 20th century, eradication of malaria seemed imminent.

But that expectation underestimated the flexibility of living creatures. Mosquitoes acquired resistance to insecticides while the parasites acquired resistance to anti-malarial drugs. Worse, the aggressive eradication campaign skipped over vast regions of the globe, especially sub-Saharan Africa.

Malaria remains a devastating problem in Africa for several reasons. Environmental conditions provide an amenable atmosphere for both Plasmodium falciparum, the most dangerous form of the parasite, and the Anopheles gambiae mosquito, the most effective vector. Also, many countries in sub-Saharan Africa lack the infrastructure to protect their citizens from malaria. Given the overwhelming scope of malarial infection in Africa, new understanding of the disease will help epidemiologists devise targeted anti-malarial strategies. [Fig pbio-0030306-g001]

**Figure pbio-0030306-g001:**
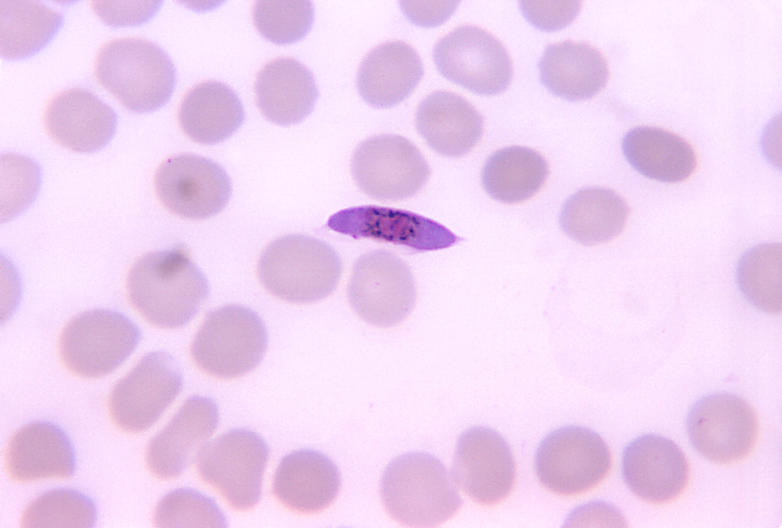
Mosquitoes are most attracted to children infected with malarial parasites in the gametocyte stage (pictured above). The Anopheles mosquito ingests gametocytes during its blood meal. (CDC/ Dr. Mae Melvin)

A new study conducted in Western Kenya by Jacob Koella and colleagues analyzed mosquito behavior to discover how it facilitates the transmission of malaria. The research determined that mosquitoes are more attracted to people infected with transmittable malaria than to either people infected with non-transmittable forms of the disease or uninfected people. To measure the attraction of the mosquitoes, the researchers set up a chamber of infected mosquitoes surrounded by tents containing the study participants. A device called an olfactometer wafted the odors of each participant toward the mosquitoes. Researchers measured which smell most attracted the hungry bugs.

This question had long stalled scientists because of contradictory and indirect evidence. Sweat, breath odor, and high body temperature all increase mosquitoes' blood lust, and no previous study had isolated the variable of malarial infection.

To control for the natural variation in how attractive mosquitoes found each participant, Koella et al. compared the number of mosquitoes that were attracted to infected people to the number of mosquitoes that were attracted to those same people after they were no longer infected. The researchers found that in general, an individual attracted more mosquitoes when infected with transmittable malaria. This demonstrates that malaria, in addition to causing fever, vomiting, headache, and sometimes death, causes more mosquito bites. The biting mosquitoes will then pick up the parasite and spread it to other people.

As another control, the researchers compared infection with a non-transmittable form of the parasite to infection with the transmittable form and to no infection. A mosquito can pick up the malaria parasite only when in its sexually reproductive stage. The transmittable parasite, known as a gametocyte, multiplies in the mosquito's belly before traveling to the mosquito's salivary glands and, eventually, to the blood of the next human victim. But the malaria parasite has a complicated life cycle that also includes non-transmittable asexual stages. Koella and colleagues found that these parasitic forms, unlike the sexually reproductive form, did not make humans more attractive to mosquitoes.

Previous to the recent study, malaria researchers had proved that mosquito biting rates greatly influence the spread of malaria. Koella and colleagues showed that the parasite itself increases these biting rates when it is ready for a new host.

